# Decoloniality in physiotherapy education, research and practice in South Africa

**DOI:** 10.4102/sajp.v77i1.1556

**Published:** 2021-05-28

**Authors:** Saul Cobbing

**Affiliations:** 1 Department of Physiotherapy, Faculty of Health Sciences, University of KwaZulu-Natal, Durban, South Africa

**Keywords:** decoloniality, South Africa, physiotherapy, education, research, practice, inequality, transformation

## Abstract

**Background:**

Historically, the profession of physiotherapy in South Africa has closely aligned itself with our former colonial master, the United Kingdom. Whilst efforts have been made in recent years to transform our profession, numerous challenges remain. An improved understanding of the topic of decoloniality is a useful and necessary way of beginning to address these challenges.

**Objectives:**

The aim of this opinion piece is to encourage further dialogue amongst South African physiotherapists working in all sectors – a dialogue that must focus on genuinely transforming our profession to be better suited to serving the majority of South Africans.

**Method:**

Global and local literature related to decoloniality is summarised for readers, followed by a closer scrutiny of how this topic relates to some of the challenges faced by the profession of physiotherapy in South Africa.

**Results:**

The evidence presented demonstrates that whilst some efforts have been made to transform South African physiotherapy, significant work and dialogue is required to bring about a true transformation of the profession.

**Conclusion:**

An honest and transparent conversation about decoloniality and transformation can assist in realising the potential of our profession, thereby improving the health and well-being of all South Africans.

**Clinical implications:**

Real engagement with this topic can assist in transforming who enters our profession, what we teach, where and why we conduct research and how we can ensure that physiotherapy practice contributes to real social justice by benefitting the majority of our population.

## Situating myself

In order to address such a politically loaded topic as decoloniality, it is essential that I first describe who I am in more detail. In the communication of any research, authors should be reflexive and position themselves, declaring their potential biases and acknowledging that one’s own traits and lived experiences have a direct influence on the ‘what’ and ‘how’ of collecting and disseminating research findings (Cutcher 2020). This is even more critical when attempting to discuss a topic such as decoloniality. I am a white, heterosexual, able-bodied, English-speaking, middle-class man. I am educated to a postgraduate level and am employed as a full-time associate professor in the Discipline of Physiotherapy at the University of KwaZulu-Natal (UKZN). These labels are important. As a result of these inherited and acquired advantages, I am a beneficiary of considerable privileges not enjoyed by the majority of my fellow South Africans. I have written about this fact in more detail previously, focusing on both the explicit and subtle daily benefits that this privilege affords me (Cobbing 2016). As an opinion piece, this article can only necessarily be seen from my own perspective. I cannot speak on behalf of my physiotherapy colleagues across South Africa, particularly those who identify as belonging to race groups that were previously subjected to sustained, state-sponsored physical and psychological violence by the colonial and apartheid projects. Language, phrases and words are important and this is a theme I will focus more on in this article. Thus, I use a phrase such as ‘subjected to physical and psychological violence’ rather than a euphemism such as ‘previously disadvantaged’, which does not even begin to describe the historical and continued suffering experienced by the majority of our population. This suffering, underscored by deep inequality and poverty, continues to manifest along race and gender lines, 27 years into our new democracy. My thinking that informed this article was forged by a week-long series of workshops on decoloniality that I attended in January 2020 (UKZN 2020). Soon after this, the coronavirus disease 2019 (COVID-19) pandemic changed the world. The words I had imagined committing to article then ricocheted around my brain for a number of months. This unplanned hiatus makes this article even more relevant. The COVID-19 pandemic has only emphasised the gross inequality in our country, underlining the urgent need for a considered contemplation of what we really want our profession to look like.

I am further privileged to work at an institution of higher education with brilliant and inspirational black, Indian and coloured (note how the racial classifications of the apartheid regime persist) physiotherapy lecturers – representing different genders, ethnicities and religious faiths. A question that may arise is why I did not choose to co-author this opinion piece with one or more of these colleagues. The simple answer is that this is my opinion only, influenced no doubt by the labels I have used to describe myself above. As much as I may have attempted to educate myself on the topic of decoloniality, I cannot pretend that I understand the lived experience of one of my black female colleagues. It is helpful if academic articles, even an opinion piece such as this, include an explicit aim. The aim of this article, then, is to encourage further dialogue amongst South African physiotherapists working in all sectors – a conversation that must focus on genuinely transforming our profession to be better suited to serving the majority of our population. Mullen (2019), a white woman writing about decolonisation in Canada, discusses how it is problematic to attempt to speak on behalf of people of other races. Conversely, it is very possible that by writing as a white person, one can ‘trouble the colonial mindset’ and make certain invisibilities visible (Mullen 2019). For the purpose of my article, the particular invisibility I would like to reveal, therefore, is what I see as our profession’s aversion to talking about issues such as transformation and decoloniality. The conversation around decoloniality has already been initiated in South Africa, led by brilliant black men and women. Sadly, it is a conversation that mostly passes by unnoticed, particularly in the health science disciplines and even more so, in our profession, where I would propose that there is far more of a focus on technical skills than on deep, critical thought. In this article, I will briefly summarise the terminology relating to decoloniality, discuss some of the work being done in this area in South Africa and then place a specific focus on how we can begin to truly transform South African physiotherapy education, research and practice. I hope that my article encourages a continued conversation, within and beyond our profession, involving individuals from all backgrounds and all occupational sectors.

## Decoloniality and related terms

This section includes a very brief summary of the important terminology that is required to better understand the ideas and recommendations proposed in my article. I would thus encourage readers to read the full works of these authors and others so as to raise one’s consciousness of the continued effects of coloniality, as well as the emerging decolonial response. South Africa has a history of colonisation stretching back over 350 years (Dye & La Croix 2020). In 1652, Jan van Riebeeck established the first permanent European settlement in Southern Africa, under a distinctively flat mountain that the indigenous Khoisan people called Hoerikwaggo or ‘the mountain in the sea’ (Landy, Belaidi & Gaudry 2017). The horrors of colonisation were compounded by the evils of formalised, state-sanctioned apartheid from 1948 until the country’s first democratic election in 1994. The term ‘decolonisation’ has gained significant traction in the South African higher education discourse in recent years and is used to describe a shift away from purely colonial knowledge and practices. Ndlovu-Gatsheni (2017a), a professor in psychology at the University of South Africa, has stated that what we gained in 1994 was democracy without decolonisation. However, in the sense that decolonisation refers literally to the removal of a colonial power (Collins Dictionary 2020), which has occurred in most cases across the globe, perhaps a more accurate term to employ in conversations around this topic is ‘decoloniality’. Decoloniality has been defined as the ‘liberation of (ex-) colonised people from global coloniality’ (Ndlovu-Gatsheni 2015:485). With regard to global knowledge structures, Grosfoguel (2013) describes a centuries-old history of epistemicide, which involved the calculated extermination of indigenous knowledge systems by the Western colonial powers. Indigenous knowledge and traditions were then replaced by the knowledge produced primarily by men from five Western countries, namely, France, Germany, Italy, the United Kingdom and the United States of America (Grosfoguel 2013). This newer, colonial knowledge is now foundational to all Western universities (including South African universities) and applies to subjects as diverse as philosophy and economics and certainly includes the health sciences. To introduce some other terms for readers who may not be au fait with educational theory, colonial expansion in Southern Africa ensured that white Western epistemology (the study of how knowledge is acquired) and white Western ontology (the study of all that exists) were favoured over indigenous knowledge and understandings of reality.

Decoloniality is a direct response to coloniality, which Maldonado-Torres (2007) has defined as persistent patterns of power resulting from colonialism that continue to define culture, social relations and knowledge production, long after the colonial power has departed. Ndlovu-Gatsheni, Maldonado-Torres and others have unpacked the term ‘coloniality’ further, in relation to three distinct concepts, namely, the coloniality of power (the continued favouring of elites even after the withdrawal of colonial administrations), the coloniality of knowledge (the demonising of traditional or indigenous, non-Western knowledge) and the coloniality of being (a denial of the ‘full humanness’ of ex-colonised people, with a lack of recognition for the significant worth of their indigenous knowledge and practices). The desired liberation from coloniality described by Ndlovu-Gatsheni (2017a), has certainly not been achieved in South Africa. In this sense, Ndlovu-Gatsheni (2017a), proposed the term ‘neo-apartheid’ rather than ‘post-apartheid’ for the period from 1994 to the present day. This term better describes the persistence of racism, inequality and the exclusion of the black majority in present-day South Africa (Ndlovu-Gatsheni 2017a), This inequality continues to define power relations and knowledge production in South African universities. How else can one begin to explain the recent debacle at Stellenbosch University, where a peer-reviewed published study queried the intellect of coloured South African women (Nieuwoudt et al. 2020)? That this article has since been retracted by the *Aging Neuropsychology and Cognition* journal should not disguise the disturbing fact that such a study was conceived, sanctioned at multiple levels and widely communicated, without any thought about how offensive it was to an already marginalised group of South Africans. A similar furore erupted at the University of Cape Town concerning an article investigating why black students are less likely to study biological sciences (Nattrass 2020). Whilst there will always be a tension between ‘academic freedom’ and basic ethical standards, it is clear that studies like this are exacerbating the long-term, deeply felt wounds experienced by black South Africans. Denying this and refusing to engage with the decolonial process will only hinder any attempts to achieve real social justice in our country.

## Decoloniality in South African health sciences

A better knowledge and understanding of psychology, which straddles the health science and humanities domains, is seen as being increasingly important in addressing the myriad health issues confronting the South African population. A number of South African academics have written about the need for the profession of psychology to transform and include decolonial approaches in both training and practice. Ratele (2016) supports the call for an Africa(n)-centred psychology. This includes an understanding that things that are particular to Africa need to be taken into account when designing teaching and community engagement practices. In so doing, Western-centred approaches have to be critically challenged and indigenous psychologies from other parts of the world should also be drawn upon. Seedat (1997) states that decolonising practices should include (amongst other things) marginal voices, liberatory modes of knowledge creation, dialogical community engagement and non-hierarchical teaching and learning approaches. The health sciences in South Africa still reflect a rigid hierarchy, with medicine being dominant over all the other health science professions. This begins in the classroom, continues in professional practice and is further evident in the allocation of research funding.

A small number of academics from the ‘allied’ or rehabilitation professions (which include physiotherapy) have been writing about decolonising health science education since the dawn of democracy in South Africa. As far back as 1997, Pillay, Kathard and Samuel (1997) stated that in order for speech-language therapy and audiology to transform, it would be vital to deconstruct these professions’ curricula, which they described as being underpinned by racist, apartheid values. Fast forward to 2015, and two of these authors have written in detail about the continued, and now more urgent, need to transform how we educate health professionals in South Africa. This need is highlighted by the continued inability of these professions to serve the majority of black South Africans (Pillay & Kathard 2015). It would be difficult to argue that this state of affairs is not similar in all of the South African health professions. Writing about the physiotherapy profession, Amosun, Maart and Naidoo (2018) echoed the call made by Pillay and Kathard (2015) for the critical transformation of health science curricula. These authors state that physiotherapy curricula in South Africa, like the majority of higher education disciplines, are still influenced by the legacy of colonial education. Similarly, Ramugondo (2015) describe how constructions of occupation in the discipline of occupational therapy are dominated by white, Western, able-bodied heterosexual, female voices.

## Challenges for physiotherapy in South Africa

The very curricula taught to South African physiotherapy students remain a challenge. I will return briefly to a personal reflection. I was brought up in the Eastern Cape province of South Africa but completed my undergraduate physiotherapy degree in the United Kingdom. On commencing an academic career at the UKZN in 2010, I was genuinely shocked at how similar the curriculum was to what I had studied in England, including the modules taught, the prescribed textbooks, the methods of assessment and where the students trained (mainly in urban hospitals). This was shocking to me because of the vastly different clinical environments and conditions that our students were exposed to compared with their British counterparts. Moffat (2012) describe how the development of physiotherapy programmes offered in former British colonies (such as South Africa and a number of other African countries) was primarily informed by the curriculum developed in the United Kingdom. It would be disingenuous to suggest that South African physiotherapy programmes have not evolved in the past decade, particularly with regard to a concerted move towards decentralised clinical training models conducted in resource-poor, often rural, communities (Blose et al. 2019). Caution must be taken, however, to ensure that this is not just a geographical shift in where we work, but also a deep, philosophical shift in the way we approach our teaching. In other words, much more still needs to be done to ensure that curricula are more reflective of our country’s population, settings and health concerns. In so doing we can, as Trede (2012) state, disrupt mainstream physiotherapy discourses that tend to counter people-centred care.

Research is crucial to inform curricula and ensure that they evolve in relation to context-specific challenges. Therefore, *what* and *where* South African physiotherapists and academics choose to study is crucial. Do we really need another study on neck pain, or do we need more studies conducted in rural areas? Rather than choosing between this false dichotomy, perhaps we need to investigate, for example, how evidence-based cervical treatment techniques can succeed in resource-poor communities. Jansen and Walters (2020) state that research can never be a value-free activity in terms of what we choose to study and what methods we employ. Indeed, research conducted in South Africa often exposes what these authors describe as the racial ‘fault lines’ in our country (Jansen & Walters 2020). Ndlovu-Gatsheni (2017b) propose that research methodology can only be decolonised if we confront its dirty history. We have to ask ourselves if the methods we use routinely in physiotherapy research adequately address the concerns faced by the majority of South Africans. In health sciences, there is a tendency to rely on quantitative, positivist methodologies (e.g. measuring range of motion or oxygen saturation) to answer research questions. If techniques that have been proven to work in the first world are not as successful in South Africa, we need to ask more profound questions to find out why this is so. Pillay et al. (1997) argued that a more critical paradigm is required to address specific South African research questions, including the use of more qualitative methodologies such as true action research. Over 20 years later, it is debatable whether this is happening in our country. A recent systematic review of qualitative research articles focusing on patient-centeredness in physiotherapy (Wijma et al. 2017) revealed common themes such as individuality and social characteristics, which are key decolonial perspectives. All 14 articles included in this review, however, were conducted in the Western world (Australasia, Europe and North America).

A third challenge facing the physiotherapy profession is that presented by the massive inequality in South Africa. This country is, according to Stats SA (2020), amongst the most unequal societies on earth, with a heavily racialised and gendered labour force. These facts have a very real impact on physiotherapy education and practice. Despite some efforts being made at transforming this profession, Amosun et al. (2018) state that the demographics of physiotherapy students at certain South African universities have not changed in any significant way (one only has to watch the students from the eight universities at the annual Comrades Marathon expo to understand this). Furthermore, the representation of black physiotherapists in the South African Society of Physiotherapy (SASP) does not match the demographic make-up of qualified South African physiotherapists. As these authors point out, this may be because of the fact that people with financial resources (often white – *my addition*) may be better placed to enter tertiary education and volunteer for their professional society than people with less means (often black – *also my addition*). A recent study by Louw et al. (2020) presents an in-depth analysis of the demographics of the physiotherapy profession between 1938 and 2018. Whilst there has been a consistent increase in the number of registrations by black, Indian and coloured physiotherapists with the Health Professions Council of South Africa over these eight decades, the demographics of the profession are still markedly different from the population at large. Black people account for 17% of all physiotherapists, despite making up 80% of the South African population. Conversely, white people make up 55% of all registered physiotherapists, more than seven times the proportion of white people in the population (8%) (Louw et al. 2020). Similar racial disparities prevail in the professions of occupational therapy (Ned et al. 2020), speech therapy and audiology (Pillay et al. 2020). These similarities with other professions should not exculpate the profession of physiotherapy from any closer examination or rebuke. Rather, this highlights how, collectively, rehabilitation professions in South Africa are failing to transform. A more positive spin on this is that this shared challenge provides an ideal opportunity to work together amongst professions to address and remedy this state of affairs.

There is a clear need to transform our physiotherapy programmes, places of practice and the SASP. Would a separate association for physiotherapists, the Physiotherapy Association of South Africa (PASA), have been created if physiotherapists of all races felt truly represented and valued? This is the type of tough question that needs to be faced, with transparency and honesty, if we are to really transform our profession. It is important that both these professional associations work together to bring about true, lasting transformation. The inequality we see at the university and professional society level is mirrored by the disparities we see between the private and public health sectors in South Africa. Whatever one’s views and concerns are on the proposed National Health Insurance (NHI), it is clear that a situation where a majority of South Africans are denied life-changing (and indeed life-saving) physiotherapy services is contrary to the right to equitable healthcare espoused in Section 27 of our Constitution (Republic of South Africa 1996). But nobody will push for greater recognition for our profession in this new proposed model, other than physiotherapists. Transforming the profession and aligning the curricula and practice more with the NHI can only strengthen the argument for the provision of more physiotherapy services in this new model.

## Charting a way forward

As mentioned earlier, it is hoped that this opinion piece introduces important issues related to the concept of decoloniality, whilst also stimulating further discussion. The purpose of this article is certainly not to disparage anybody or cause friction in an already fractured country. If you have carefully read what I have written you will see that there are as many questions here as there are proposed solutions. I want, therefore, to open up a conversation among all concerned physiotherapists in South Africa. As discussed, there is a need to transform physiotherapy, education and practice. Whilst we do have critical European influences that have shaped our profession, we must always remember that we are situated in Africa. Ndlovu-Gatsheni (2017a) describes how we need to go through a process of ‘learning to unlearn in order to relearn’. We need to thus interrogate what we have been taught and strive to learn more about our own country and communities in order to see Africa as a viable base from which we can frame and make sense of our research, teaching and practice. As an example of this, the study by Louw et al. (2020) described above provides a fascinating analysis of the demographics of South African physiotherapists. These authors do, themselves, acknowledge that this analysis is limited without further research into the lived experiences of physiotherapists, relating to the transformation of physiotherapy education and practice. It must also be observed that the above figures only relate to race. More granular statistics relating to characteristics such as social class, schooling, place of origin (rural or urban), sexuality and description of disability are other factors that should be considered if we are to truly transform our profession. It is not possible in this article to do full justice to the very complex issue of achieving both demographic and pedagogical transformation in our profession. I hope, however, that therapists and academics reading this can add to this conversation in the form of more in-depth explorations of this particular question.

Cooper and Ratele (2018) delve into a discussion of how the black consciousness of Steven Bantu Biko can ensure that their profession of psychology can serve *all* of humanity. Similarly, as South African physiotherapists, we need to ensure that we improve the lives of all South Africans rather than continue to support educational and professional practices that mainly cater to a white minority and emerging black elite. Biko was a promising medical student in Durban, but his dream of a bright future in healthcare was never realised because of his political consciousness: a consciousness that ultimately led to his murder at the hands of the apartheid police at the age of 30. In addition to his immense political legacy, Biko also left us with these words: ‘[*i*]n time, we shall be in a position to bestow on South Africa the greatest possible gift – a more human face’ (Ahluwalia & Zegeye 2001). Is it not time, then, that we truly commit to the task at hand; that of decolonising and transforming the profession of physiotherapy in South Africa? Not because it is the expedient or trendy thing to do, but because we genuinely *see* the faces of every South African and value their humanity. As an academic, I am well aware that this responsibility starts with myself. Pillay (2020) highlights the inconvenient truth that academics are too often distracted by the commodification of academia (publish or perish) to become activists for social change, in any meaningful way. In other more affluent countries, academics may indeed have the luxury to write what they like (apologies to Biko) but in South Africa, the privilege of our profession and the platform we are given as academics demands that we translate our work into tangible change.

In advocating for a postmodernist approach to physiotherapy research, we should employ critical thinking to reconstruct physiotherapy curricula from the bottom-up (Nicholls 2012), whilst also developing models of practice that are far more diverse and inclusive (Nicholls & Holmes 2012). Decolonised curricula and pedagogies can ensure a closer alignment for black students, who have often felt alienated from higher learning in South Africa, between who they are and what they are learning (Vorster & Quinn 2017). By truly committing to this task, South African educators can, as Zembylas (2018) phrases it, ‘reclaim humanity in knowing and knowledge-making’. Transforming curricula is the responsibility of all individuals involved in physiotherapy education and something that, on a personal level, I am driving both in terms of the modules that I teach and in my role as academic leader of my department. Practical examples of this include the introduction of African-centred philosophy and indigenous knowledge content into a second year module I teach (which is the first physiotherapy module our students engage with). At a department level we have moved towards a decentralised clinical training model in the final two years of study. More important perhaps than the geographical shift to rural hospitals and clinics that this model entails is the pedagogical and philosophical shift that we are employing, teaching students to involve local communities in all aspects of their work. In this model, a lay community healthcare worker, for example, is seen as being as vital to a patient’s well-being as a surgeon or an intensive care unit nurse.

## Conclusion

South Africa is a country of great potential yet suffers from gross inequality that manifests itself along racial lines and is a grim reminder of our violent history. The profession of physiotherapy can assist in bringing out this potential by improving the health and quality of life of all South Africans. In order to achieve this, however, we need to take a closer look at the methods of education, research and practice in our profession. In this opinion piece, I have discussed some of the key concepts related to decoloniality and presented some of the challenges facing physiotherapy in South Africa. I would encourage readers of this article to delve into the works of the authors listed here, particularly those writing from a local perspective. In doing so, I hope that a dialogue is sparked and maintained that focuses on genuinely transforming our profession for the benefit of all physiotherapists and the people that we serve.
